# Serum uric acid predictive value and prognostic impact in rheumatoid arthritis’ associated interstitial lung disease

**DOI:** 10.3389/fmed.2025.1623557

**Published:** 2025-08-05

**Authors:** Qiongwen Hu, Xiaocheng Cheng, Ying Lan, Huxin Lei, Changchun Niu, Yang Luo

**Affiliations:** ^1^Chongqing Medical University, Chongqing, China; ^2^Department of Clinical Laboratory, Chongqing General Hospital, Chongqing University, Chongqing, China; ^3^Department of Respiratory and Critical Care Medicine, The First Affiliated Hospital of Chongqing Medical University, Chongqing, China; ^4^Intensive Care Unit, Affiliated Hospital of Chengdu University, Chengdu, China

**Keywords:** rheumatoid arthritis, interstitial lung disease, uric acid, predictive value, prognostic impact

## Abstract

**Importance:**

It is critical to identify novel biomarkers for the prediction and prognosis of rheumatoid arthritis-associated interstitial lung disease (RA-ILD).

**Objective:**

The objective of the study was to investigate the predictive and prognostic significance of serum uric acid (UA) in RA-ILD.

**Design, setting, and participants:**

In this multicenter retrospective cohort study, demographic data, medical history, and laboratory results of 829 RA patients were extracted from electronic medical records between December 2018 and January 2024. The cohort was divided into an RA-ILD group (*n* = 351) and an RA-no-ILD group (*n* = 478).

**Main outcomes and measures:**

The primary endpoint was the predictive capacity of baseline UA levels for the occurrence of ILD in RA patients. The secondary endpoints were all-cause mortality and the rehospitalization rate in RA-ILD patients.

**Results:**

The univariate analysis identified elevated levels of UA as a significant risk factor for the development of ILD in RA patients (OR [95% CI]: 1.68 [1.23–2.28], *p* = 0.001). The multivariate analysis confirmed that elevated levels of UA remained an independent risk factor. The subgroup analysis demonstrated a stronger predictive utility of elevated UA levels in younger RA patients (age < 65), particularly those with shorter disease duration and no comorbidities (OR [95% CI]: 3.66 [1.66–8.05], *p* = 0.001; AUC_ROC_ = 0.718). During follow-up, RA-ILD patients with elevated UA levels exhibited significantly higher all-cause mortality (22.1% vs. 13.1%; HR [95% CI]: 1.80 [1.03–3.17], *p* = 0.040) and rehospitalization rates (51.9% vs. 44.4%; HR [95% CI]: 1.41 [1.00–1.98], *p* = 0.047) compared to those with lower UA levels.

**Conclusion and relevance:**

Elevated levels of serum UA may serve as a predictive marker for ILD development in RA patients, particularly in younger individuals without comorbidities, and a prognostic indicator for increased mortality and rehospitalization rates in RA-ILD patients.

**Clinical trial registration:**

ClinicalTrials.gov, identifier NCT06036537.

## Introduction

Rheumatoid arthritis (RA) is a systemic autoimmune disease characterized by chronic synovial inflammation and progressive joint destruction, often accompanied by extra-articular manifestations. Among these comorbidities, rheumatoid arthritis-associated interstitial lung disease (RA-ILD) has emerged as a key determinant of mortality, although its clinical significance remains underrecognized (1, 2). Epidemiologic studies estimate that 5–10% of RA patients develop clinically significant ILD, with mortality rates 2- to 10-fold higher compared to RA patients without pulmonary involvement (3). Current diagnostic biomarkers, including anti-citrullinated protein antibodies (ACPA), MUC5B promoter polymorphisms, and Krebs von den Lungen-6 (KL-6), demonstrate limited prognostic utility for stratifying disease progression and survival outcomes (4). This diagnostic inadequacy underscores the urgent need to identify novel predictive biomarkers for ILD development in RA populations and elucidate prognostic determinants in established RA-ILD cases. Addressing these knowledge gaps may facilitate early intervention strategies that attenuate pulmonary deterioration and improve survival rates in this high-risk cohort.

Uric acid (UA), a crystallizable metabolite of purine catabolism, functions as an endogenous damage-associated molecular pattern (DAMP) that activates the NLR family pyrin domain containing 3 (NLRP3) inflammasome through crystal-dependent and independent mechanisms (5). Mechanistic studies by Gasse et al. demonstrated that UA exacerbates pulmonary inflammation and fibrotic remodeling through TLR2/TLR4-mediated NLRP3 inflammasome activation in alveolar macrophages, establishing a molecular link between hyperuricemia and lung pathology (6). Notably, the NLRP3 inflammasome has been pharmacologically validated as a therapeutic target in RA, where its aberrant activation drives synovitis and bone erosion, while targeted inhibition significantly ameliorates disease severity in preclinical models (7, 8). These pathophysiological connections suggest that UA may serve as a dual modulator influencing both ILD development in RA patients and clinical trajectories in established RA-ILD. Supporting this hypothesis, Andersen et al. conducted a retrospective cohort study involving 212 ILD patients and identified serum UA levels exceeding the 90th percentile as an independent predictor of ILD-related mortality (9). Nevertheless, the specific prognostic impact of hyperuricemia on RA-ILD mortality remains uncharacterized. This study aims to address two critical knowledge gaps: (1) Whether elevated levels of UA constitute a novel risk factor for ILD incidence in RA populations and (2) how UA dynamics modulate clinical outcomes in RA-ILD patients.

## Materials and methods

### Study design

We conducted a multicenter retrospective cohort study registered under clinicaltrials.gov (identifier NCT06036537). We selected cases for this study using strict inclusion and exclusion criteria based on a continuous sampling method at the First Affiliated Hospital of Chongqing Medical University and Chongqing General Hospital between December 2018 and January 2024. Patients with a confirmed RA diagnosis were enrolled. The exclusion criteria were as follows: (1) Age <18 years, (2) treatment with uric acid-lowering drugs, (3) systemic autoimmune diseases other than RA, such as systemic lupus erythematosus (SLE), ANCA-associated vasculitis, primary Sjogren’s syndrome (pSS), systemic sclerosis (SSc), and myositis, (4) cancer, (5) pulmonary tuberculosis, and (6) advanced chronic kidney disease or severe loss of kidney function, defined as an estimated glomerular filtration rate (eGFR) less than 30 mL/min/1.73 m^2^, which significantly impairs the excretion of metabolic substances (10, 11). This study was approved by the Institutional Ethics Board of Chongqing General Hospital (approval NO. KY S2023-024-01). All methods were performed following the relevant guidelines and regulations.

### Data collection

We consecutively collected electronic medical records of patients hospitalized for the first time between December 2018 and January 2024 to obtain their demographic characteristics, medical history, RA disease duration, laboratory test results, comorbidities, and treatment information. These data were collected independently by two investigators and then checked by another two investigators. RA-ILD patients were followed up via telephone by two investigators until the end of August 2024.

### Definition and study outcomes

Confirmed RA cases were classified in accordance with the 2010 American College of Rheumatology/European League Against Rheumatism (ACR/EULAR) classification criteria. The diagnosis of interstitial lung disease (ILD) in RA patients was consecutively confirmed through a multidisciplinary consensus review of high-resolution computed tomography (HRCT) findings, in accordance with the Fleischner Society guidelines (12). The study participants were stratified into hyperuricemic and normouricemic groups based on an optimized serum UA cutoff value derived from receiver operating characteristic (ROC) curve analysis for ILD prediction. The Youden index-derived cutoff maximized both sensitivity and specificity for discriminating ILD. The primary endpoint was the predictive capacity of baseline UA levels for incident ILD in RA patients. The secondary endpoints were all-cause mortality and rehospitalization rates in RA-ILD patients. RA-ILD patients were prospectively enrolled in a longitudinal observational cohort and were followed up through August 2024. The exposure period for mortality was defined as the number of days from study inclusion to death or the end of follow-up. The exposure period for rehospitalization was defined as the number of days from study inclusion to the first rehospitalization within 6 months.

### Statistical analysis

Sample size determination was conducted using power analysis for logistic regression with a single binary predictor variable, employing the Wald test for calculating the odds ratio (OR) (13). The calculation assumed 80% power to detect a clinically meaningful effect size at the 0.05 significance level (two-tailed), yielding a minimum sample requirement of 745 participants. Categorical variables were described as frequency rates and percentages. Continuous variables were described as mean (standard deviation: [SD]) if normally distributed and as median (interquartile range: [IQR]) if not normally distributed. The χ^2^ test was performed to assess differences in the categorical variables between the two groups. A *t*-test or Mann–Whitney U test was performed to compare the continuous variables, depending on whether they were normally distributed. Binary and multiple logistic regression analyses were performed to estimate the odds ratio (OR) for risk factors. The Kaplan–Meier product-limit estimation method was used to estimate survival rates of RA-ILD patients. Univariate and multivariate Cox proportional hazards models were used to estimate hazard ratios (HRs) and 95% confidence intervals (CIs). To investigate whether UA provides additional value in predicting ILD in RA patients, we performed receiver operating characteristic (ROC) curve analysis and assessed the equality of the area under the curve (AUC). We considered that a *p*-value of < 0.050 is statistically significant, and all *p*-values were three-tailed. Statistical analyses were performed using R software (version 4.3) and SPSS software (version 22.0).

## Results

### Patients

**Figure 1:**
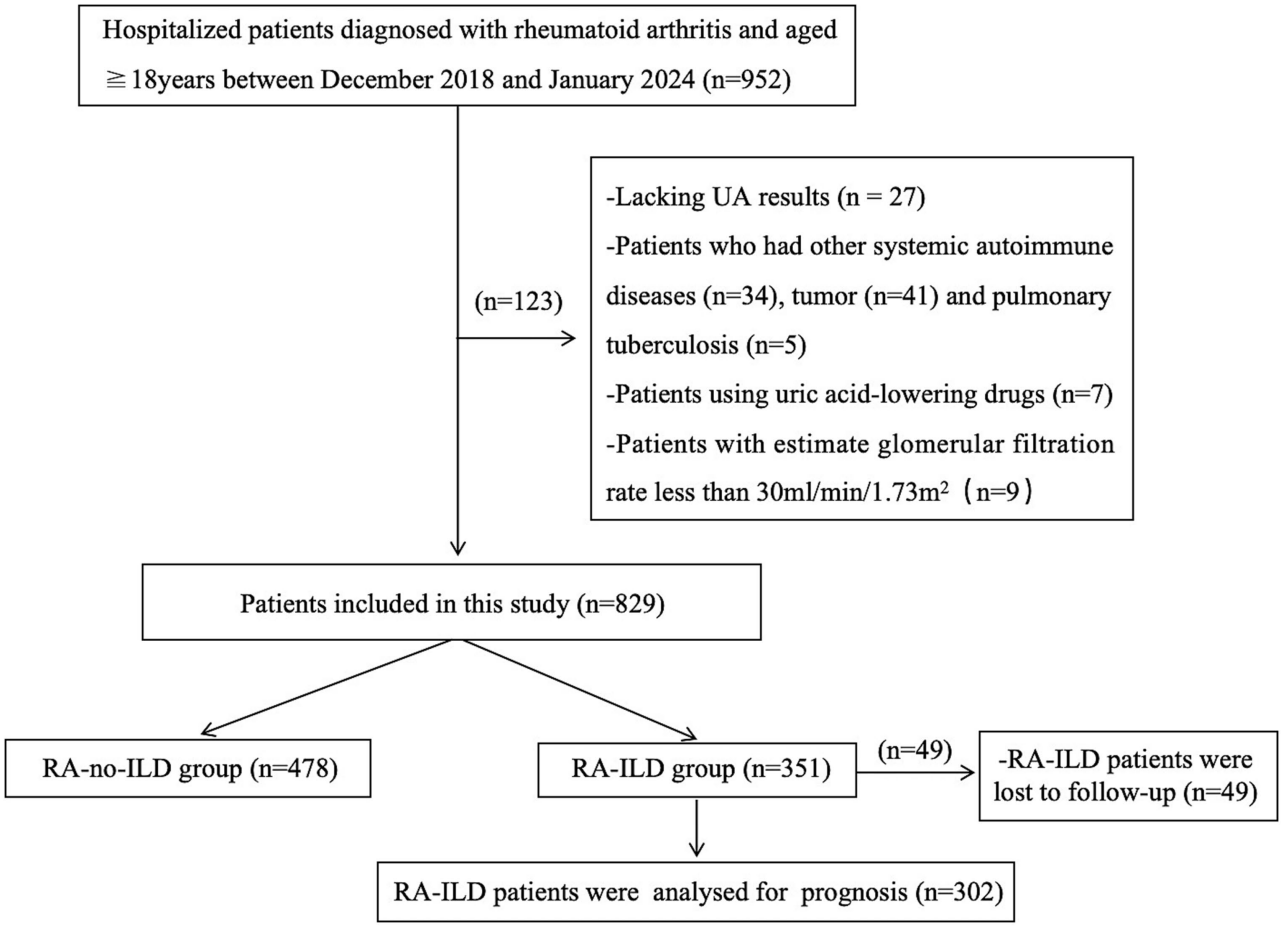
Flow diagram of patient recruitment.

The study initially enrolled 952 consecutive hospitalized patients with RA. Patients without UA data (*n* = 27) were excluded. Patients who had other systemic autoimmune diseases (*n* = 34), tumors (*n* = 41), and pulmonary tuberculosis (*n* = 5) were also excluded. In addition, patients who used uric acid-lowering drugs (*n* = 7) and those with an eGFR less than 30 mL/min/1.73 m^2^ (*n* = 9) were excluded. Finally, a total of 829 patients were included in the study. The patients were divided into two groups: RA-ILD group (351 patients) and RA-no-ILD group (478 patients). A total of 351 patients with RA-ILD were followed up by telephone, and 49 of them were lost to follow-up. Finally, 302 RA-ILD patients were analyzed for all-cause mortality and rehospitalization rates (Figure 1).

Significant sex disparity was observed between the cohorts, with a female predominance in the RA-no-ILD patient group (78.87% [95% CI 75.2–82.3%]) compared to the RA-ILD patient group (54.70% [95% CI 49.5–59.8%]; χ^2^ = 48.7, *p* < 0.001). Age distribution differed significantly between the groups, demonstrating a mean difference of 4.17 years (RA-no-ILD: 59.76 ± 13.01 years vs. RA-ILD: 63.93 ± 11.19 years; t = 5.32, *p* < 0.001). Biochemical analysis revealed clinically significant intergroup variations in UA levels (RA-no-ILD: 277.20 ± 92.00 μmol/L vs. RA-ILD: 293.60 ± 97.80 μmol/L; mean difference 16.4 μmol/L, *p* = 0.010). Comprehensive baseline characteristics, including inflammatory markers and serological profiles, are presented in Table 1.

**Table 1:** Baseline characteristics of the included patients.

Characteristics	All (*n* = 829)		*p*-value
	RA-ILD (*n* = 351)	RA-no-ILD (*n* = 478)	
General demographics			
Sex (female)	192 (54.70%)	377 (78.87%)	<0.001
Age (mean ± SD, years)	63.91 ± 11.28	60.19 ± 12.59	<0.001
Smoking	97 (27.64%)	95 (19.87%)	0.009
RA duration (IQR, months)	72 (24, 120)	60 (12, 132)	0.312
Laboratory results			
RF (IU/mL)	197.00 (34.43, 609.50)	81.35 (20.55, 287.50)	<0.001
Anti-CCP (RU/mL)	122.00 (17.85, 347.06)	85.75 (11.57, 200.00)	0.003
ESR (mm/h)	66.00 (43.00, 94.75)	48.00 (25.00, 75.00)	<0.001
DAS28	6.75 (5.64, 7.63)	5.83 (4.55, 7.10)	<0.001
CRP (mg/L)	20.60 (8.50, 65.20)	11.30 (3.74, 39.61)	<0.001
UA (μmol/L)	293.60 ± 97.80	277.20 ± 92.00	0.010
Urea (mmol/L)	5.50 (4.30, 6.80)	5.10 (4.10, 6.30)	0.008
Crea (μmol/L)	62.00 (53.00, 75.00)	57.00 (49.00, 67.00)	<0.001
ALP (U/L)	78.00 (65.00, 98.55)	79.50 (66.00, 97.00)	0.897
ALT(U/L)	16.00 (10.50, 23.00)	14.00 (10.00, 21.00)	0.056
AST (U/L)	18.85 (15.00, 25.00)	18.00 (14.00, 22.80)	0.004
WBC (10^9^/L)	7.32 (5.85, 9.54)	6.24 (4.92, 7.82)	<0.001
NEUT (%)	70.53 ± 11.24	67.74 ± 10.74	0.001
LYMPH (%)	18.65 (14.25, 24.62)	21.40 (16.10, 27.70)	<0.001
MONO (%)	6.70 (5.30, 8.50)	6.90 (5.30, 8.70)	0.962
PLT (10^9^/L)	245.50 (186.75, 316.00)	235.00 (188.00, 301.00)	0.294
HGB (g/L)	120.00 (103.00, 132.00)	118.00 (103.00, 130.00)	0.394
TC (mmol/L)	4.22 (3.48, 4.72)	4.24 (3.62, 5.02)	0.104
TG (mmol/L)	1.14 (0.87, 1.53)	1.11 (0.84, 1.52)	0.320
HDL-C (mmol/L)	1.15 (0.95, 1.39)	1.25 (1.00, 1.55)	0.001
LDL-C (mmol/L)	2.51 (2.05, 3.13)	2.47 (1.97, 3.01)	0.383
APOE(mg/dL)	2.22 (1.94, 2.52)	2.59 (2.08, 4.33)	<0.001
Lp(a) (mg/L)	175.00 (67.00, 358.00)	162.00 (80.50, 419.00)	0.624
PCT (ng/mL)	0.07 (0.05, 0.18)	0.06 (0.03, 0.15)	0.106
IL-6 (pg/mL)	20.91 (7.26, 67.76)	20.94 (4.64, 63.83)	0.569
D-DIMER (mg/LFEU)	1.43 (0.61, 3.12)	0.91 (0.28, 2.34)	<0.001
FIB (g/L)	4.59 (3.72, 5.92)	4.13 (3.14, 5.33)	<0.001
TP (g/L)	68.00 (62.25, 73.25)	68.00 (64.00, 74.00)	0.372
ALB (g/L)	37.00 ± 5.33	38.72 ± 5.66	<0.001
GLB (g/L)	30.00 (26.00, 35.55)	29.00 (26.00, 33.00)	0.053
Cys-C (mg/L)	1.14 (0.97, 1.44)	1.07 (0.90, 1.31)	0.002
eGFR (mL/min/1.73 m^2^)	98.90 (85.03, 109.00)	97.70 (86.54, 109.00)	0.706
Current treatments			
GCs use	191 (54.42%)	73 (15.27%)	<0.001
ISD use	294 (83.76%)	251 (52.51%)	<0.001
Diuretics use	15 (4.27%)	9 (1.88%)	0.043
Concomitant diseases			
HTN	138 (39.32%)	158 (33.05%)	0.063
DM	68 (19.37%)	69 (14.44%)	0.059
CLD	53 (15.10%)	39 (8.16%)	0.002
CAD	33 (9.40%)	53 (11.09%)	0.432

ALB, Albumin; ALP, Alkaline phosphatase; ALT, Alanine aminotransferase; Anti-CCP, Anti-cyclic citrulline polypeptide antibody; APOE, Apolipoprotein E; AST, Aspartate aminotransferase; CLD, Chronic lung disease; CRP, C-reactive protein; CAD, Coronary artery disease; Cys-C, Cystatin C; DAS28, Disease Activity Score 28; DM, Diabetes mellitus; ESR, Erythrocyte sedimentation rate; eGFR, Estimate glomerular filtration rate; FIB, Fibrinogen; GCs, Glucocorticoids; GLB, Globulin; HDL-C, High density lipoprotein cholesterol; HGB, Hemoglobin; HTN, Hypertension; ISD, Immunosuppressive drug; IL-6, Interleukin 6; Lp(a), Lipoprotein a; LDL-C, Low density lipoprotein cholesterol; LYMPH, Lymphocyte percentage; MONO, Monocyte percentage; NEUT, Neutrophil percentage; PCT, Procalcitonin; PLT, Platelet count; RF, Rheumatoid factor; TC, Total cholesterol; TG, Triglyceride; TP, Total protein; UA, Uric acid; WBC, White blood cell count.

### Logistic regression analysis of risk factors for incident ILD in RA patients

Univariate logistic regression analysis was performed to determine risk factors for ILD incidence in RA patients. The analysis showed that female sex, age, smoking, rheumatoid factor (RF), erythrocyte sedimentation rate (ESR), disease activity score 28 (DAS28), C-reactive protein (CRP), high UA, aspartate aminotransferase (AST), white blood cell count (WBC), neutrophil ratio (NEUT %), fibrinogen (FIB), albumin (ALB), globulin (GLB), cystatin C (Cys-C), glucocorticoids (GCs) use, immunosuppressive drug (ISD) use, diuretics use, and chronic lung disease (CLD) were all statistically significant risk factors for the incidence of ILD in RA patients (Table 2).

**Table 2:** Univariate logistics regression analysis of risk factors for incident ILD in RA patients.

General demographics	Odds ratio (95% CI)	*p*-value
General demographics		
Sex (female)	3.098 (2.303–4.169)	<0.001
Age (IQR, years)	1.028 (1.017–1.040)	<0.001
Smoking	1.544 (1.037–2.299)	0.032
RA duration (month)	1.000 (0.998–1.001)	0.461
Laboratory results		
RF (IU/mL)	1.000 (1.000–1.001)	<0.001
Anti-CCP (RU/mL)	1.000 (1.000–1.000)	0.828
ESR (mm/h)	1.014 (1.009–1.018)	<0.001
DAS28	1.237 (1.133–1.351)	<0.001
CRP (mg/L)	1.004 (1.002–1.007)	0.002
High UA* (μmol/L)	1.676 (1.230, 2.283)	0.001
Urea (mmol/L)	1.042 (0.992–1.094)	0.101
Crea (μmol/L)	1.004 (0.998, 1.010)	0.173
ALP (U/L)	1.003 (0.995, 1.011)	0.430
AST (U/L)	1.016 (1.002–1.030)	0.023
WBC (10^9^/L)	1.166 (1.108–1.226)	<0.001
NEUT (%)	1.027 (1.014–1.041)	<0.001
LYMPH (%)	0.959 (0.943–0.975)	<0.001
MONO (%)	1.014 (0.984–1.044)	0.374
PLT (10^9^/L)	1.001 (0.999–1.002)	0.384
HGB (g/L)	1.002 (0.998–1.007)	0.282
CHO (mmol/L)	0.890 (0.773–1.024)	0.103
TG (mmol/L)	0.986 (0.858–1.134)	0.847
HDL-C (mmol/L)	1.013 (0.978–1.049)	0.482
LDL-C (mmol/L)	1.134 (0.969–1.326)	0.117
APOE (mg/dL)	0.968 (0.936–1.002)	0.062
Lp(a) (mg/L)	1.000 (0.999–1.000)	0.721
PCT (ng/mL)	1.260 (0.810–1.960)	0.305
IL-6 (pg/mL)	1.000 (0.997–1.003)	0.900
D-DIMER (mg/LFEU)	1.034 (0.984–1.087)	0.189
FIB (g/L)	1.146 (1.047–1.255)	0.003
TP (g/L)	0.998 (0.990–1.005)	0.540
ALB (g/L)	0.947 (0.923–0.972)	<0.001
GLB (g/L)	1.027 (1.005–1.050)	0.015
Cys-C (mg/L)	1.422 (1.063–1.901)	0.018
eGFR (mL/min/1.73 m^2^)	0.993 (0.986–1.000)	0.064
Current treatments		
GCs use	3.990 (2.806–5.672)	<0.001
ISD use	0.555 (0.246–1.247)	0.154
Diuretics use	2.326 (1.006–5.379)	0.048
Concomitant diseases		
HTN	0.782 (0.584–1.048)	0.100
DM	0.837 (0.575–1.218)	0.353
CLD	1.249 (0.693–2.252)	0.459
CAD	0.495 (0.311–0.790)	0.003

ALB, Albumin; ALP, Alkaline phosphatase; ALT, Alanine aminotransferase; Anti-CCP, Anti-cyclic citrulline polypeptide antibody; APOE, Apolipoprotein E; AST, Aspartate aminotransferase; CRP, C-reactive protein; CLD, Chronic lung disease; CAD, Coronary artery disease; Cys-C, Cystatin C; DAS28, Disease Activity Score 28; DM, Diabetes mellitus; eGFR, Estimate glomerular filtration rate; ESR, Erythrocyte sedimentation rate; FIB, Fibrinogen; GCs, Glucocorticoids; GLB, Globulin; HGB, Hemoglobin; HTN, Hypertension; IL-6, Interleukin 6; ISD, Immunosuppressive drug; LDL-C, Low density lipoprotein cholesterol; Lp(a), Lipoprotein a; LYMPH, Lymphocyte percentage; MONO, Monocyte percentage; NEUT, Neutrophil percentage; PCT, Procalcitonin; PLT, Platelet count; RF, Rheumatoid factor; TP, Total protein; TC, Total cholesterol; TG, Triglyceride; UA, Uric acid; WBC, White blood cell count; *, UA ≥ 227.00 μmol/L.

**Figure 2:**
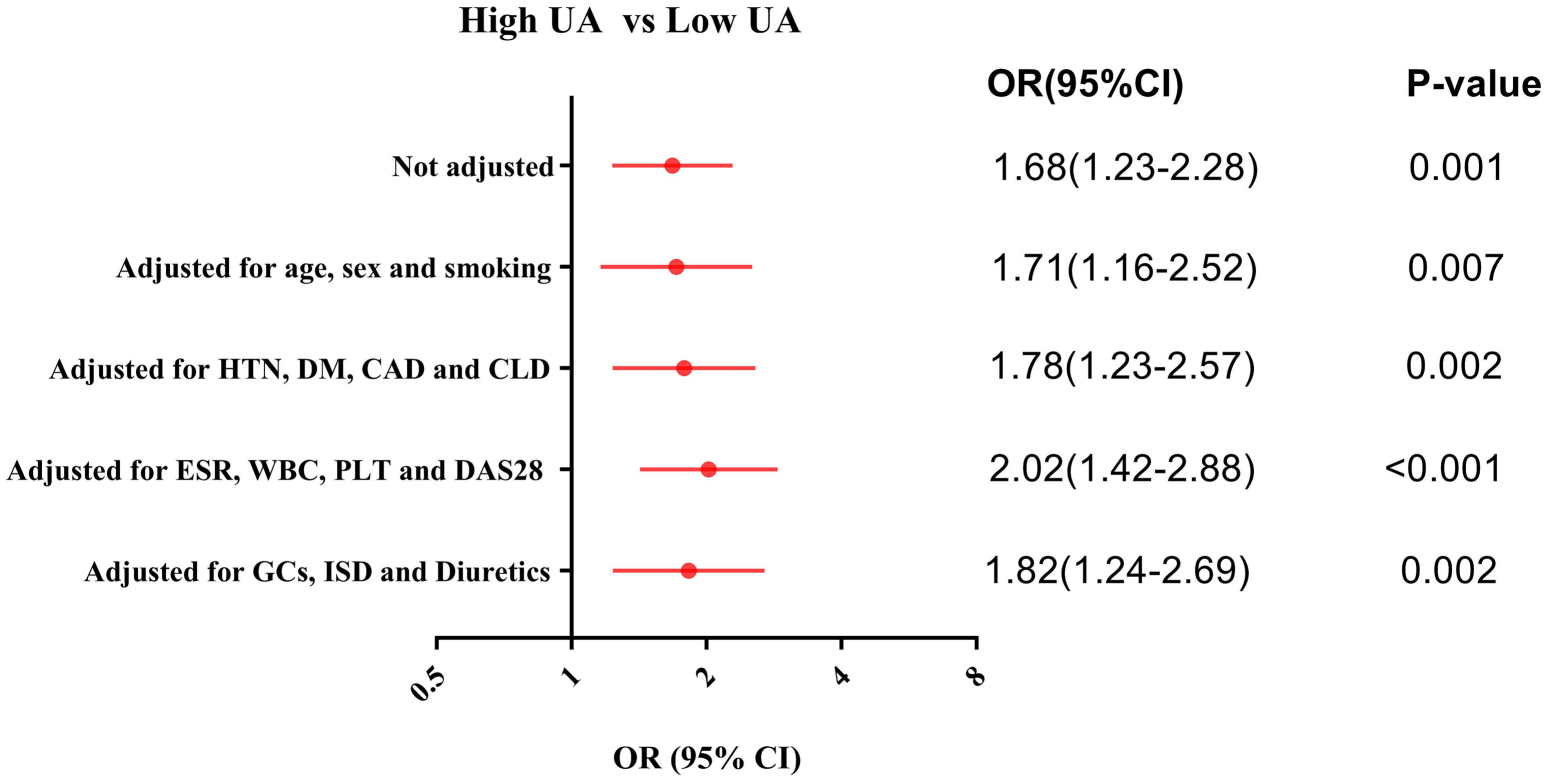
Logistic regression adjusted analysis for the risk of incident ILD in the RA patients with high UA. CAD, Coronary artery disease; CLD, Chronic lung disease; DAS28, Disease Activity Score 28; DM, Diabetes mellitus; ESR, Erythrocyte sedimentation rate; GCs, Glucocorticoids; HTN, Hypertension; ISD, Immunosuppressive drug; PLT, Platelet count; WBC, White blood cell count.

Patients with high uric acid levels were defined as those with a serum UA concentration ≥ 227.00 μmol/L upon admission, according to the ROC curve–derived cutoff for ILD prediction. The univariate logistic regression analysis showed that high levels of UA was a risk factor for incident ILD in RA patients (OR [95% CI]: 1.68 [1.23–2.28], *p* = 0.001). To rule out potential confounding variables affecting the results, we performed multivariate logistic regression analyses with sequential adjustments for the following factors: (1) Baseline characteristics: age, sex, and smoking (adjusted OR [95% CI]: 1.71 [1.16–2.52], *p* = 0.007); (2) comorbidities: hypertension (HTN), diabetes mellitus (DM), coronary artery disease (CAD), and chronic lung disease (CLD) (adjusted OR [95% CI]: 1.78 [1.23–2.57], *p* = 0.002); (3) inflammatory markers: ESR, WBC, PLT, and DAS28 (adjusted OR [95% CI]: 2.02 [1.42–2.88], *p* < 0.001); and (4) medications: glucocorticoids (GCs), immunosuppressive drug (ISD), and diuretics use (adjusted OR [95% CI]: 1.82 [1.24–2.69], *p* = 0.002). After adjustment for these covariates, elevated UA levels remained an independent risk factor for incident ILD in RA patients (Figure 2).

### Subgroup analysis of the effect of UA levels on the risk of incident ILD in RA patients

**Figure 3:**
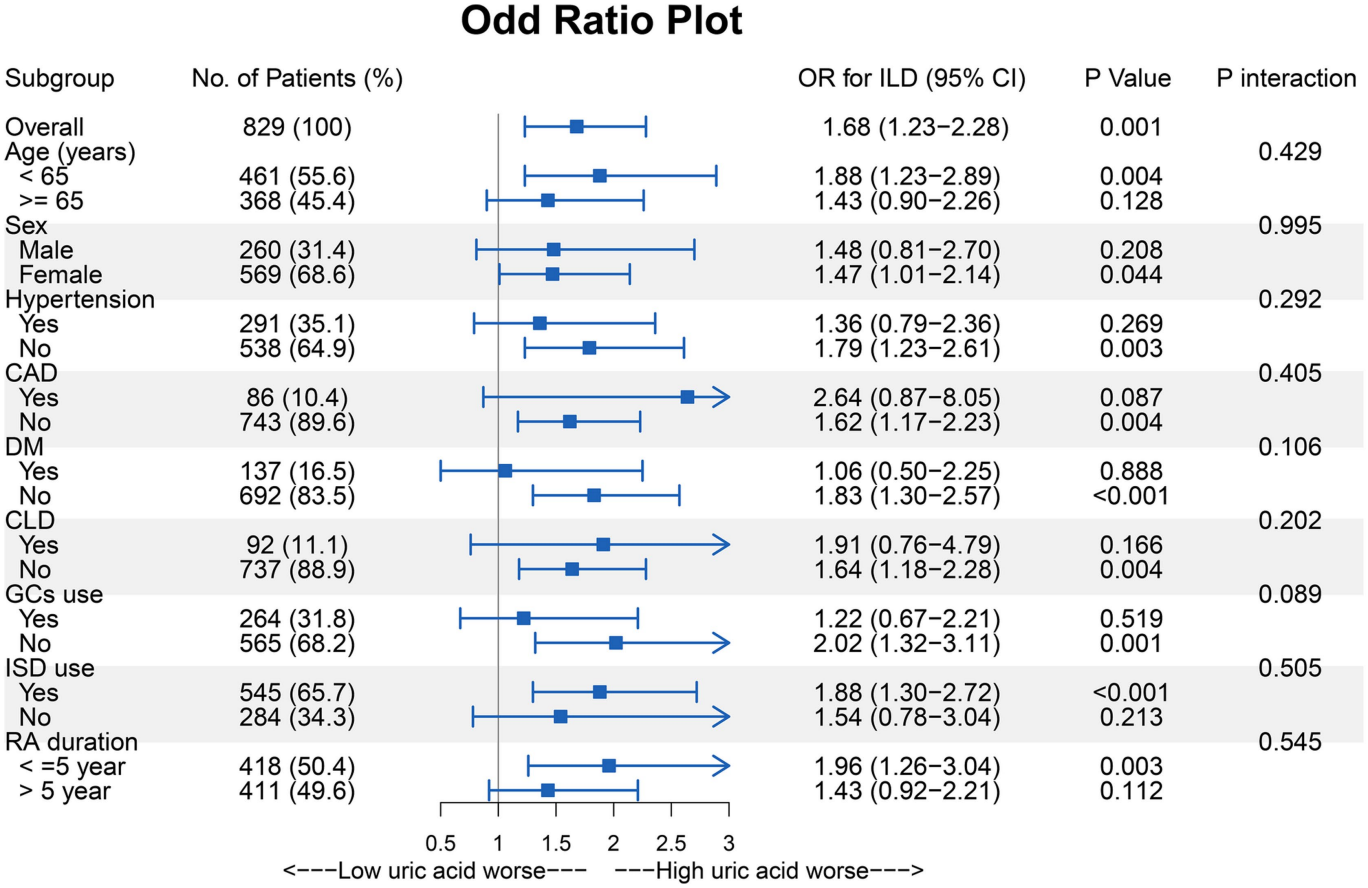
Subgroup analysis of the effect of UA on the risk of incident ILD in the RA patients. CAD, Coronary artery disease; CLD, Chronic lung disease; DM, Diabetes mellitus; GCs, Glucocorticoids; ISD, Immunosuppressive drug.

To further investigate the relationship between UA and RA-ILD, subgroup analyses of several variables were performed. Although there were no statistical differences between the groups, it appeared that UA for the prediction of RA-ILD tended to be of greater value in patients who were younger, had shorter disease duration, and had fewer comorbidities (Figure 3).

**Figure 4:**
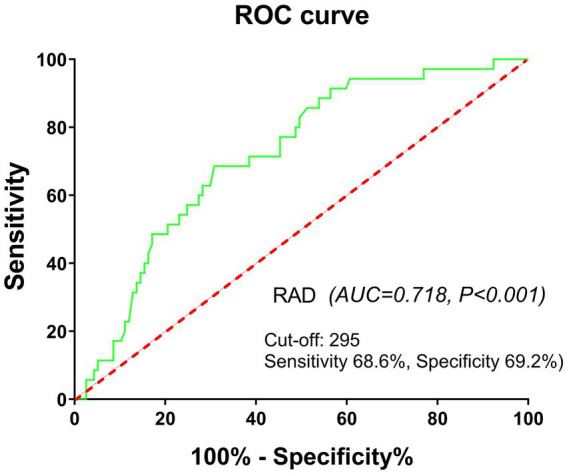
ROC curve analysis showing significant power of UA for ILD prediction in a refined RA cohort. Refined RA cohort: age <65 years, absence of concomitant diseases, disease duration ≤5 years, and glucocorticoid-naïve status.

Therefore, we established a refined RA cohort (*n* = 152) based on stringent inclusion criteria: age <65 years, absence of comorbidities (HTN, DM, CAD, CLD), disease duration ≤5 years, and glucocorticoid-naïve status. In this homogeneous subpopulation, elevated UA levels demonstrated enhanced predictive capacity for incident ILD development (OR [95% CI]: 3.66 [1.66–8.05], *p* = 0.001). Further validation using ROC curve analysis revealed significant discriminative power of UA for ILD prediction (AUC = 0.718, *p* < 0.001), with an optimal cutoff value of 295.00 μmol/L (sensitivity 68.6%, specificity 69.2%) (Figure 4).

### Cumulative survival curves of all-cause mortality and rehospitalization rates

**Figure 5:**
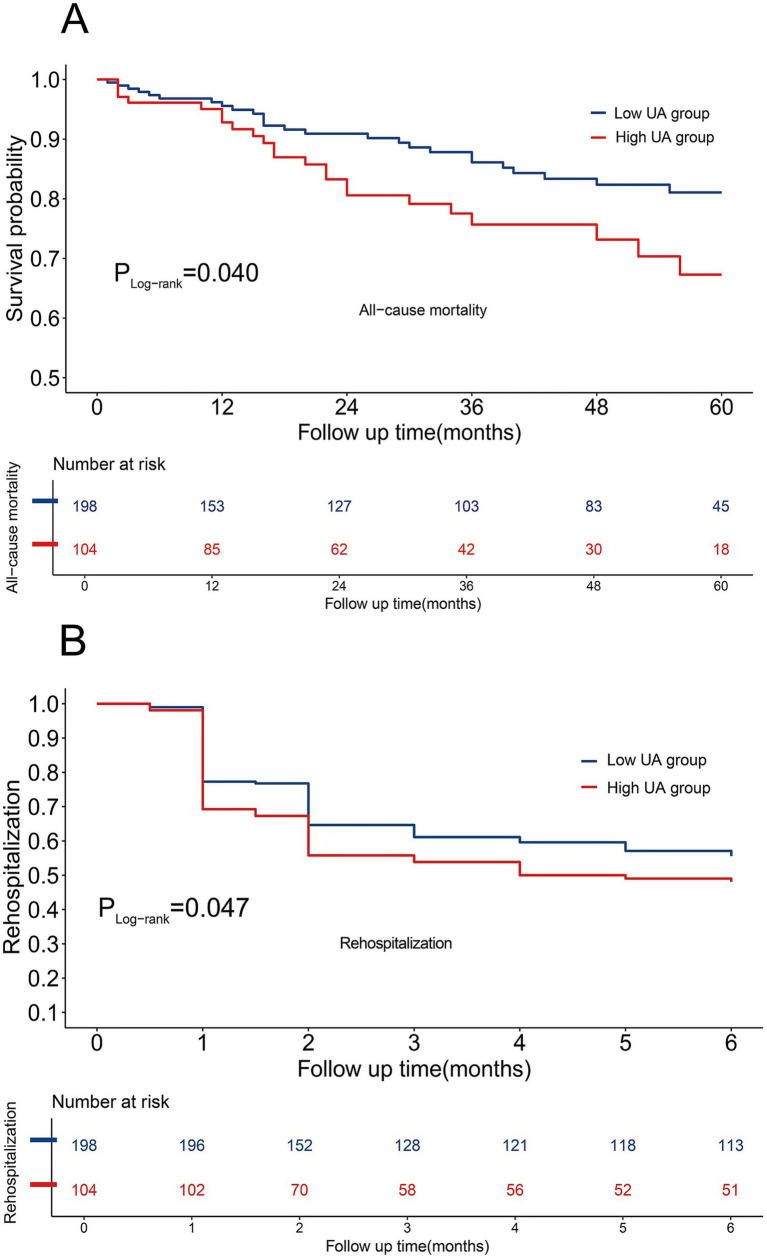
**(A)** Cumulative survival curves of all-cause mortality. **(B)** Cumulative survival curves of rehospitalization rates.

The cohort comprised 302 RA-ILD patients who completed follow-up, demonstrating distinct mortality and rehospitalization trajectories: the mean follow-up duration for mortality was 34.0 ± 21.1 months and 4.0 ± 2.3 months for rehospitalization. All-cause mortality occurred in 49 patients (16.2%), with a significant between-group disparity in crude incidence rates (high-UA: 22.1% [23/104] vs. low-UA: 13.1% [26/198]). The Cox proportional hazards models revealed an elevated mortality risk in hyperuricemic patients (HR [95%CI]: 1.80 [1.03, 3.17], *p* = 0.040). The rehospitalization analysis demonstrated a similar risk elevation (high-UA: 51.9% [54/104] vs. low-UA: 44.4% [88/198]), corresponding to an elevated rehospitalization risk in hyperuricemic patients (HR [95% CI]: 1.41 [1.00–1.98], *p* = 0.047). Temporal risk patterns were visualized using stratified Kaplan–Meier curves (Figures 5A,B).

## Discussion

Our investigation yielded four principal findings: (1) RA-ILD patients exhibited significantly elevated serum UA concentrations compared to RA-no-ILD counterparts; (2) hyperuricemia was an independent predictor of incident ILD in RA patients after multivariable-adjusted analyses; (3) this association was particularly strong in younger RA patients with shorter disease duration and no comorbidities; and (4) importantly, elevated UA levels was associated with an increased risk of mortality and rehospitalization in the RA-ILD cohort.

The enzymatic machinery for purine catabolism, particularly xanthine oxidase-mediated UA generation, demonstrates ubiquitous tissue distribution including pulmonary parenchyma, where it serves as the terminal pathway for purine nucleotide degradation. Preclinical evidence suggests that hyperuricemia may exacerbate RA autoimmune pathogenesis through dual mechanisms: crystal-dependent NLRP3 inflammasome activation and crystal-independent enhancement of Th17 differentiation (14). Mechanistically, genetic polymorphisms in inflammatory mediators significantly modulate UA homeostasis. For instance, Patel et al. reported that RA patients harboring the TGF-β1 + 869 T allele (rs1982073)exhibited 28% higher serum UA concentrations and heightened systemic inflammation compared to wild-type counterparts—findings potentially mediated through enhanced TGF-β1 signaling and impaired UA renal excretion (15). Clinico-pathological correlations further reveal that sustained UA elevation may perpetuate chronic inflammation through positive feedback loops involving CRP (*r* = 0.42, *p* < 0.001), TNF-*α* (*r* = 0.38, *p* = 0.002), and IL-6 (*r* = 0.35, *p =* 0.005), suggesting that hyperuricemia serves as a biomarker and a pathogenic indicator in RA-related systemic inflammation (16).

ILD, a prevalent yet underrecognized extra-articular manifestation of RA, contributes substantially to disease-related morbidity and mortality (17, 18). Emerging evidence indicates that diagnostic delays in RA-ILD correlate with accelerated mortality rates, underscoring the critical need for early detection strategies (19). While established risk factors, including tobacco exposure, advanced age at RA onset, and severe extra-articular manifestations, have been identified (20), novel predictive biomarkers remain underexplored. Our multicenter cohort study involving 829 RA patients found a statistical difference in serum UA levels between RA-ILD and RA-no-ILD patients. Multivariate logistic regression analyses demonstrated that hyperuricemia independently predicted ILD development after comprehensive adjustment for baseline characteristics (age, sex, and smoking), comorbidities (HTN, DM, CAD, and CLD), inflammatory markers (ESR, WBC, PLT, and DAS28), and medications (GCs, ISD, and diuretics). However, the absolute difference in UA levels between the two groups was less than 1 mg/dL. This minor discrepancy may be related to the higher comorbidity burden and the widespread use of antirheumatic medications in the included patient cohort.

Current evidence regarding UA’s role in RA-ILD pathogenesis remains sparse, with only Wang et al.’s small sample case–control study (*n* = 162 RA-ILD vs. 104 RA-no-ILD) preliminarily suggesting UA’s predictive potential for RA-ILD development (21). While concordant with our findings, our multicenter cohort study advances this evidence through three critical dimensions: (1) enhanced statistical power via expanded enrollment (N = 829); (2) demonstration of UA’s independence as a risk factor after multivariable adjustment for inflammatory markers, DAS28, concomitant diseases, and other confounders; and (3) identification of a high-risk subpopulation in which hyperuricemia confers a 3.66-fold increased risk of ILD (95%CI 1.66–8.05, *p* = 0.001)—specifically, RA patients aged <65 years, with disease duration <5 years, and without major comorbidities. This amplified effect in the high-risk subpopulation likely reflects minimized confounding from polypharmacy and preserved endogenous purine metabolism integrity, whereas geriatric patients’ metabolic dysregulation and multidrug regimens may attenuate UA’s biomarker fidelity through pharmacokinetic interactions and cumulative oxidative burden.

Current evidence identifies several prognostic determinants of RA-ILD progression, including advanced age, male sex, elevated DAS28, a usual interstitial pneumonia (UIP) pattern on HRCT, extensive pulmonary involvement, and anti-cyclic citrulline polypeptide (CCP) antibody titers (12). Risk stratification models incorporating age ≥60 years and HRCT radiomics features have demonstrated robust predictive accuracy for 5-year mortality (22). Despite these advances, the pathophysiological role of UA in RA-ILD remains insufficiently characterized. While Wang et al. (21) established significant inverse correlations between serum/bronchoalveolar lavage fluid (BALF) UA levels and forced vital capacity, our study extends this paradigm by elucidating UA’s dual clinical relevance (1) as an independent predictor of incident ILD in RA patients across multivariable-adjusted models and (2) as a prognostic biomarker for mortality and rehospitalization in established RA-ILD. Mechanistically, UA-mediated NLRP3 inflammasome activation induces IL-1β overproduction, perpetuating pulmonary inflammation through fibroblast-to-myofibroblast transition and extracellular matrix deposition—a pathway corroborated in murine models of silica-induced lung fibrosis (6).

This investigation has several limitations that warrant acknowledgment. Firstly, the retrospective design introduces potential selection bias and residual confounding despite multivariable adjustments. Secondly, while all-cause mortality and rehospitalization rates provide clinically relevant endpoints, the absence of serial pulmonary function tests (e.g., FVC% predicted) and quantitative imaging biomarkers (e.g., HRCT fibrosis scores) limits the precise stratification of ILD progression severity. Thirdly, the exclusive enrollment of East Asian participants restricts external validity, highlighting the need for validation in multiethnic cohorts due to known ethnic disparities in urate-handling polymorphisms. Fourthly, due to incomplete medical records of outpatients, we only included newly hospitalized RA patients, which introduces selection bias that limits the applicability of the results to outpatients with more stable conditions. Finally, the enhanced UA prognostic utility observed in patients aged <65 years, with disease duration of <5 years, and without comorbidities represents an exploratory finding requiring confirmation through prospective trials powered for subgroup interactions.

## Conclusion

A major insight from our data is that elevated levels of serum UA may serve as a predictive marker for ILD development in RA patients, particularly in younger individuals without comorbidities, and a prognostic indicator for increased mortality and rehospitalization in RA-ILD. Further prospective studies are warranted to validate these findings and explore the underlying mechanisms.

## Data Availability

The original contributions presented in the study are included in the article/supplementary material, further inquiries can be directed to the corresponding authors.
